# Metformin synergistic pemetrexed suppresses non‐small‐cell lung cancer cell proliferation and invasion in vitro

**DOI:** 10.1002/cam4.1133

**Published:** 2017-07-18

**Authors:** Yan Zhang, Xiuli Feng, Tao Li, Erpan Yi, Yu Li

**Affiliations:** ^1^ Departments of Respiratory Medicine Qilu hospital of Shandong University Jinan Shandong China; ^2^ Departments of Respiratory Medicine People's Hospital of Qingzhou Weifang Shandong China

**Keywords:** Combination, metformin, NSCLC, pemetrexed

## Abstract

The aim of this study was to investigate whether metformin in combination with pemetrexed has an effect on the treatment of non‐small‐cell lung cancer (NSCLC) models and to explore the related molecular mechanism. The half maximal inhibitory concentration (IC50) and combination index (CI) of metformin and pemetrexed were detected by the CCK8 assay to assess the antiproliferative and therapeutic effects of the two‐drug combination. Flow cytometry (FCM) and invasion assays were used to estimate the variation in apoptosis rate and invasion ability of the differently treated NSCLC cell lines. Apoptotic markers were detected by western blotting to validate the data related to the antiproliferation and proapoptosis effects. Metformin monotherapy inhibited the growth of NSCLC cell lines and reduced the invasion ability to different degrees compared with the control groups (*P* < 0.05). Metformin in combination with pemetrexed produced a synergistic effect (CI < 0.90) compared with the two drugs in monotherapy in the three tested NSCLC cell lines. Metformin in combination with pemetrexed significantly increased the cell numbers of HCC827 cells at S phase (*P* < 0.001), and the combination therapy had no influence on the A549 and H1975 cell lines. We found that combining metformin with pemetrexed induced more cell apoptosis than metformin or pemetrexed used alone (*P* < 0.05), which was validated by the apoptotic markers. These results demonstrate that the combination of metformin and pemetrexed has a synergistic effect on the treatment of NSCLC cell lines by inducing apoptosis or blocking the cell cycle. Our data indicate that the combination of metformin and pemetrexed could have beneficial antitumor effects on NSCLC cells in vitro.

## Introduction

Lung cancer is one of the most common causes of tumor‐related mortality and a tremendous health challenge worldwide 1. Eighty‐five percent of lung cancer patients are affected by non‐small‐cell lung cancer (NSCLC); among these NSCLC cases, adenocarcinoma accounts for the most frequent histology 2, 3. When diagnosed, patients who are at stage IIIB~IV and can no longer undergo surgery 4 have to accept traditional cytotoxic chemotherapy and radiotherapy. At present, platinum‐based systemic chemotherapy is most commonly used in clinical treatment, including platinum/paclitaxel, platinum/gemcitabine, cisplatin/docetaxel, cisplatin, or carboplatin/pemetrexed. Among them, pemetrexed has the least toxicity and best tolerability compared with the other cytotoxic agents 5. Numerous studies have found that the combination of the platinum and pemetrexed regimens could be well tolerated, relatively prolong the overall survival (OS) of patients and be used as a front‐line therapy for patients with advanced lung adenocarcinoma 6, 7. However, clinical studies have demonstrated that pemetrexed had difficulty improving the patient survival rate compared with other chemotherapy drugs, such as docetaxel. Therefore, the overall effect is disappointing 5, 8. Searching for a new auxiliary medicine to enhance chemotherapy effects and improve survival rates for lung adenocarcinoma patients has been a hotspot of current research.

Metformin is a well‐tolerated biguanide agent that is widely used as a first‐line drug by more than 120 million patients with type 2 diabetes mellitus worldwide 9. However, since the 1970s, Dilman has found that biguanide antidiabetes medicine had antisenile and anticancer effects 10. Metformin has attracted increasing attention for its antitumor role. Retrospective and epidemiologic studies have suggested that metformin is related to a declined risk of cancer incidence in patients with type 2 diabetes and improved their survival 11, 12, 13. Laboratory studies have shown that metformin inhibits lung cancer cell proliferation and induces apoptosis 14, 15, 16. Moreover, animal xenograft experiments suggest that metformin prevents lung tumorigenesis 17 and significantly inhibits tumor growth in mice 16. The antitumor effects of metformin suggest that it can be used as a new drug in combined chemotherapy for the clinical treatment of lung cancer. Metformin has been investigated in combination with chemicals such as cisplatin and etoposide in lung cancer cells 18, 19, but studies combined with pemetrexed for lung adenocarcinoma have not yet been reported.

In our research, we hypothesize that metformin may play a role as an auxiliary or inhibitor to regulate cancer cell growth in vitro. Therefore, this study examined whether metformin in combination with pemetrexed can cause synergism, summation or antagonism in the treatment of NSCLC in vitro, which would represent a new treatment option for patients with lung adenocarcinoma.

## Materials and Methods

### Cell lines and reagents

The human NSCLC cell lines A549, HCC827, and H1975 were purchased from the Cell Bank of the Chinese Academy of Sciences (Shanghai, China) and were cultured as a monolayer in RPMI 1640 media (HyClone, Thermo Fisher Scientific Inc., UT; HCC827 and H1975) or DMEM media (HyClone; 5 mmol/L glucose, A549) supplemented with 10% fetal bovine serum (FBS, Gibco, Thermo Fisher Scientific Inc., NY), 100 U/mL penicillin (HyClone), and 100 *μ*g/mL streptomycin (HyClone) in a 37°C humidified atmosphere containing 5% CO_2_. Metformin (1,1‐dimethylbiguanide monohydrochloride; Sigma‐Aldrich, St. Louis, MO) was dissolved in phosphate‐buffered saline (PBS) at a concentration of 1 mol/L and was stored at −20°C. Pemetrexed was provided by Qilu Pharmaceutical Co., LTD. (Shandong, China) and was dissolved in normal saline (NS) at a concentration of 10 mmol/L and was finally stored at −20°C. The two drugs were diluted by complete medium for each assay. The apoptotic markers Bcl‐2 and Bax were purchased from Cell Signaling Technology (Beverly, MA).

### Cell proliferation assays

Cancer cells were seeded in 96‐well microtiter plates in 100 *μ*L of medium, and six parallel wells were assigned to each group, as well as a negative control (without cells). After 24 h of incubation, the cells in each per well were treated with different doses of metformin (0, 2.0, 4.0, 8.0, 10.0, or 20.0 mmol/L), pemetrexed (0, 0.5, 1.0, 2.0, 5.0, 10.0, or *μ*mol/L), or both (IC50 of Met + Pem 0,0.5,1.0,2.0,5.0,or 10.0 *μ*mol/L) for 48 h. After adding 10 *μ*L of CCK‐8 agent (BestBio, Shanghai, China) to each well and incubating for 2 h in the dark in an incubator at 37°C, the absorbance values (OD values) at 450 nm for all tested wells were measured using a microplate reader (Bio‐Rad) following the manufacturer's instructions. Briefly, the inhibitory and viability rates of each cell line with different treatments were calculated by comparing the OD values of the experimental groups to that of the empty group, and the half maximal inhibitory concentration (IC 50) of the two used drugs that could inhibit growth by 50% were calculated from the dose–response curves of metformin and pemetrexed alone using the Bliss method. The results of the combined treatment (combination index, CI) were analyzed using the CalcuSyn software program according to the Chou–Talalay equation, and then the value of the combination index (CI) for the combination treatment was quantitatively defined the effect, with CI < 1 (synergism), CI = 1 (additivity), and CI > 1 (antagonism).

### Assessment of apoptosis

Cancer cells were seeded in 6‐well plates and then were treated with the IC50 of metformin at 48 h (12 mmol/L for A549 cells, 5 mmol/L for HCC827 and H1975 cells), IC50 of pemetrexed at 48 h (2 *μ*mol/L for A549 and HCC827 cells, 3.5 *μ*mol/L for H1975 cells), or with the combinations of both Met and Pem. Treatment was conducted for 48 h to analysis. Briefly, the treated cells (trypsinized) were washed twice with PBS, resuspended in binding buffer at a density of 1 × 10^7^cells/mL and then were stained using an annexin V‐FITC‐PI apoptosis detection kit (BestBio, China) for 20 min at room temperature in the dark according to the manufacturer's protocols. Thereafter, the labeled cells were detected by flow cytometry and were analyzed using FlowJo 7.6 ico software.

### Cell cycle analysis

Cells subjected to the apoptosis assay were collected and washed twice with cold PBS and then were fixed with prechilled 70% ethanol for 24 h at 4°C. The fixed cells were washed and resuspended in 200 *μ*L of PBS plus 20 *μ*L of RNase A in a water bath at 37°C for 30 min. Next, the cells were filtered and incubated with 300 *μ*L of propidium iodide at 4°C for 30 min in the dark. The stained cells were detected by flow cytometry and were analyzed using ModFit LT 4.1 software.

### Migration assay

The cell matrigel migration assay was performed by 24‐well plates inserted with transwell chambers (8‐*μ*m pore size, Corning Incorporated). Next, 2 × 10^4^ cells resuspended in serum‐free RPMI 1640 or DMEM were added in the upper wells supplemented with metformin and/or pemetrexed as previously indicated and then were incubated with 10% FBS in the lower chambers. After culture for 24 h, cells on the upper chamber were lightly erased with cotton swab, and cells that had migrated into the lower bottom chamber were immobilized with 4% cold formaldehyde and were stained with 0.1% crystal violet. Next, the cells still on the lower surface of the chambers were counted in 5 random, 100 ×  microscope objective fields using a light microscope (Olympus, Tokyo, Japan).

### Western blot analysis

Following treatment, cells were collected and lysed with radioimmunoprecipitation assay (RIPA) buffer containing PMSF for 30 min on ice and then were centrifuged at 13,000*g* at 4°C for 20 min to remove insoluble fragments. The concentration of each treated group was detected using a BCA Protein Assay Kit (Beyotime, Shanghai, China) according to the manufacturer's instructions. Thereafter, the total proteins of each together with the correct dose of loading buffer were heated at 99°C for 5 min, added to 10% SDS‐PAGE gels to be electrophoretic separated and then were transferred onto polyvinylidene difluoride membranes (PVDF; Millipore, Billerica, MA). Specific primary antibodies (anti‐Bcl‐2 antibody and anti‐Bax antibody, 1:1000 dilution) and a rabbit anti‐goat immunoglobulin (IgG)‐horseradish peroxidase (HRP)‐conjugated secondary antibody (1:5000 dilution) were utilized. The control for equal protein loading was assessed using an anti‐*β*‐actin antibody (1:2000 dilution; Cell Signaling Technology, Danvers, MA). Protein bands were visualized via FluorChen E Chemiluminescent enhanced chemiluminescence (ECL) and were analyzed using the Western Blot Imaging System (Cell Biosciences, Santa Clara, CA), followed by measurement of the density of each band using Image J software (National Institutes of Health).

### Statistical analysis

All data were expressed as the means ± SD from three independent experiments unless stated otherwise. Data analysis was carried out by *T* test (pairwise comparison) and one‐way ANOVA (≥three groups comparison) using JMP^®^ 13 (SAS Institute Inc., Cary, NC), and statistical significance was evaluated at a *P* < 0.05.

## Results

### Effect of metformin and pemetrexed alone or in combination on NSCLC cell proliferation

**Figure 1:**
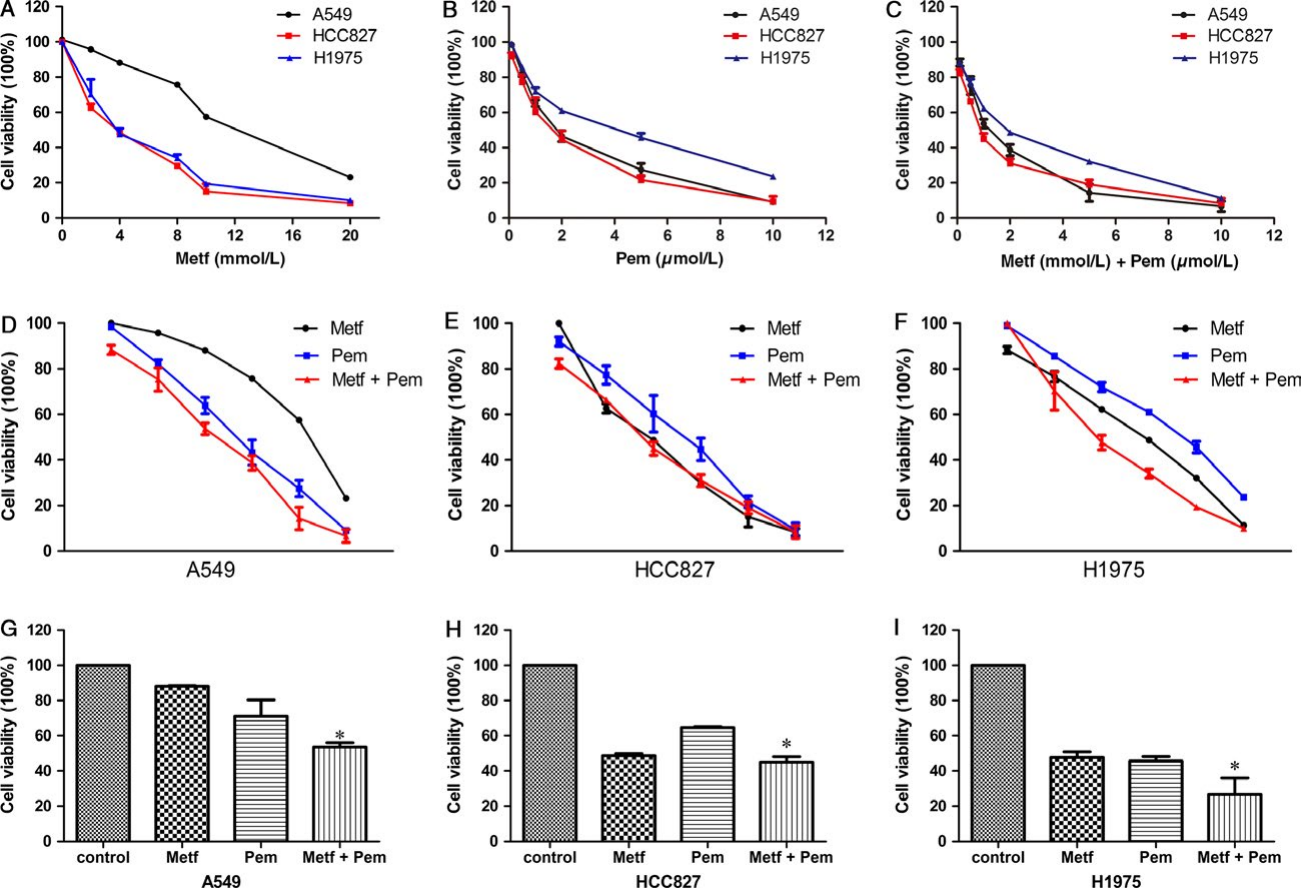
Effects of metformin and (or) pemetrexed on the proliferation of human NSCLC cell lines. (A) Antiproliferative effects of metformin on NSCLC cells. (B) Effects of pemetrexed on the proliferation of the three tested cell lines. (C) Metformin in combination with pemetrexed inhibits the proliferation of the tested cells. (D–F) Effects of metformin and (or) pemetrexed on the proliferation of A549(D), HCC827(E), and H1975(F) cell lines. (G–I) Strengthening antiproliferative effects of metformin in combination with pemetrexed on A549(G), HCC827(H), H1975(I) cell lines,*P < 0.05. Metf represents metformin; Pem represents pemetrexed.

To evaluate the potential tumor suppression or properties of metformin as in previous studies 11, 14 and to study the potential interaction between pemetrexed and metformin, we performed the CCK8 assay on a panel of three NSCLC cell lines (A549, HCC827, H1975). Our results showed that metformin alone significantly decreased the proliferation of the three NSCLC cell lines (Fig. 1A) in a concentration‐dependent manner. In Table 1, the IC50 values of metformin for the A549, HCC827, and H1975 cell lines were 11.92 ± 0.11 mmol/L, 4.72 ± 0.14 mmol/L, and 5.41 ± 0.55 mmol/L, respectively, over 48 h. Our data indicated that the effect of metformin alone on the HCC827 and H1975 cell lines was much higher than that on the A549 cell line (*P* < 0.0001), whereas there were no differences between the HCC827 and H1975 cell lines (*P* > 0.05). Similarly, in Figure 1B, pemetrexed also dose dependently suppressed the proliferation of cells with, IC50 values of 1.82 ± 0.17, 1.54 ± 0.30, and 3.37 ± 0.14 *μ*mol/L at 48 h for the A549, HCC827, H1975 cell lines, respectively (Table 1). The sensitivity of the H1975 cell line to pemetrexed was slightly lower than that of the other two cell lines (*P* < 0.05). The combination of metformin at IC50 and different dose of pemetrexed significantly enhanced cell proliferation inhibition of A549 and HCC827 compared with that of H1975 (Fig. 1C). In Figure 1D, G, F, and I, for A549 and H1975 cells, the antiproliferative effect of the combination group was significantly increased compared with that of metformin and pemetrexed alone. For HCC827 cells, the antiproliferative effect of the combination treatment was only stronger than that of the pemetrexed‐treated group (Fig. 1E and H). In addition, metformin in combination with pemetrexed resulted in robust inhibition of cell proliferation versus the two drugs alone, with ½ CI values of 0.56, 0.63, and 0.64 (Table 1, CI < 0.9) for A549, HCC827, and H1975, respectively. Thus, the combination treatment of metformin and pemetrexed is suggested to cause synergistic effects on the proliferation of NSCLC.

**Table 1:** The IC50 values and Combination index (CI) in each cell line with different drugs tested

	Met (mmol/L)	Pem (*μ*mol/L)	½ CI	CI
A549	11.92 ± 0.11^a^	1.82 ± 0.17	0.56	0.50
HCC827	4.72 ± 0.14	1.54 ± 0.30	0.63	0.49
H1975	5.41 ± 0.55	3.37 ± 0.14^b^	0.64	0.53

a The IC50 of Met on A549 cell line was significantly higher than that on HCC827 and H1975 cell line *P* < 0.05

b The IC50 of Pem on H1975 cell line was significantly higher than that on A549 and HCC827 cell line *P* < 0.05

### Effects on apoptosis by treatment with metformin and pemetrexed or both

**Figure 2:**
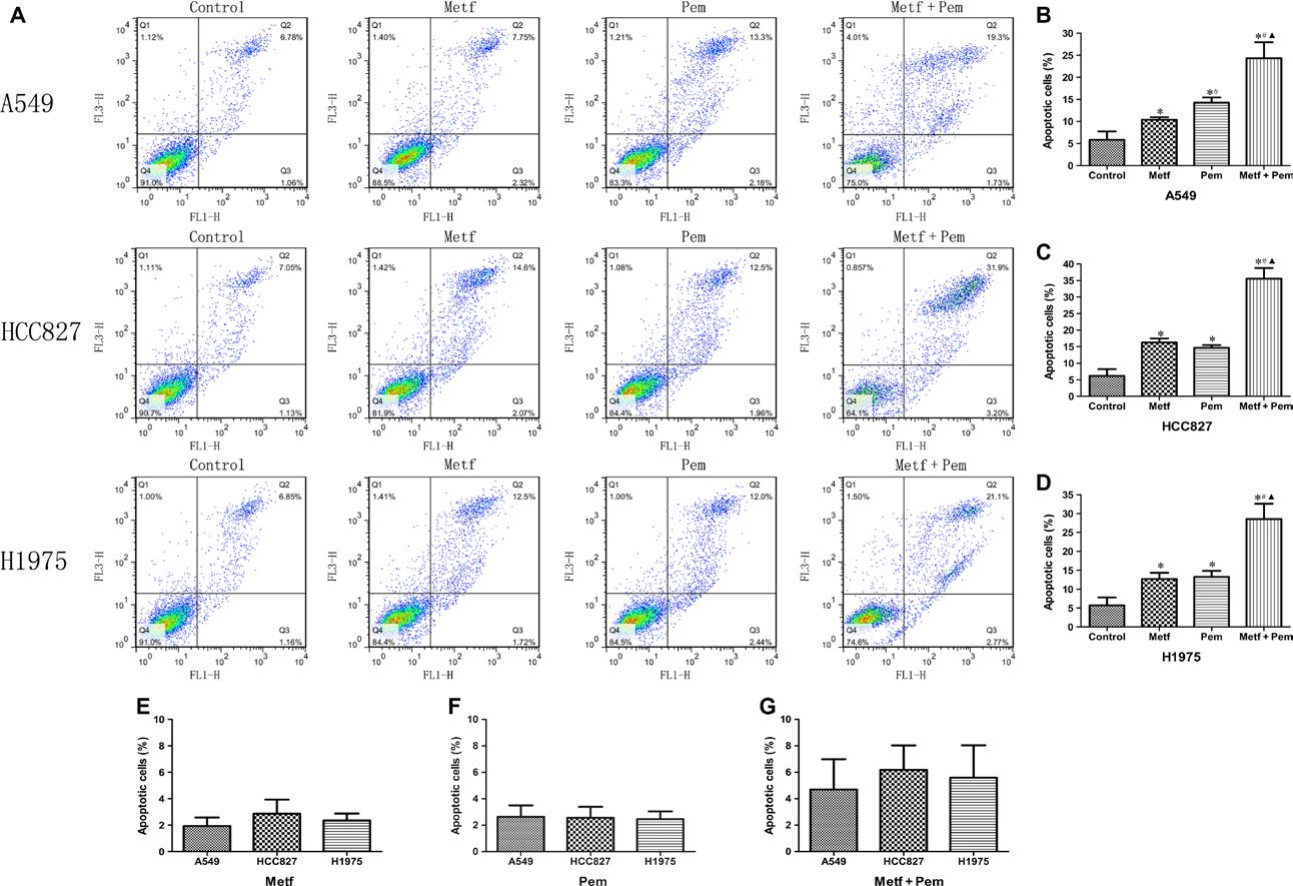
Metformin in combination with pemetrexed significantly strengthens the apoptosis of the three tested NSCLC cell lines. (A) Representative apoptosis results of A549, HCC827, and H1975 cell lines under different treatments. (B‐D) Quantitative analysis of cell apoptosis in A549, HCC827, H1975 cell lines treated with metformin, pemetrexed and the combination. (E‐G) Metformin and pemetrexed alone or the combination group separately showed no difference on A549, HCC827, H1975 cell lines. *P < 0.05 compared with the corresponding control group; ^#^ *P* < 0.05 compared with Metf alone; ^▲^ *P* < 0.05 compared with Pem alone.

In Figure 2A–D, we found that apoptosis rates of the A549, HCC827, and H1975 cell lines treated by metformin were 10.40 ± 0.57%, 16.28 ± 1.21%, and 12.68 ± 1.67%. The apoptosis rates of the A549, HCC827, and H1975 cell lines treated by pemetrexed were 14.26 ± 1.17%, 14.65 ± 0.84%, and 13.22 ± 1.60%. Metformin and pemetrexed alone enhanced apoptosis of the A549, HCC827, and H1975 cell lines compared with that of the control group. The apoptosis rates of the A549, HCC827, and H1975 cell lines treated by the combination therapy were 24.34 ± 3.62%, 35.55 ± 3.25%, and 28.54 ± 4.07%. The combination therapy had a further synergistic effect on enhancing apoptosis of the three NSCLC cell lines compared with the single drug treatment (*P* < 0.001). In Figure 2E, there were no obvious differences in cell apoptosis among the A549, HCC827, and H1975 cells treated with metformin alone as well as pemetrexed alone (Fig. 2F). Similarly, for the combination treatment, there were no obvious differences in cell apoptosis among A549, HCC827, and H1975 cells (Fig. 2G).

### Cell cycle regulation

**Figure 3:**
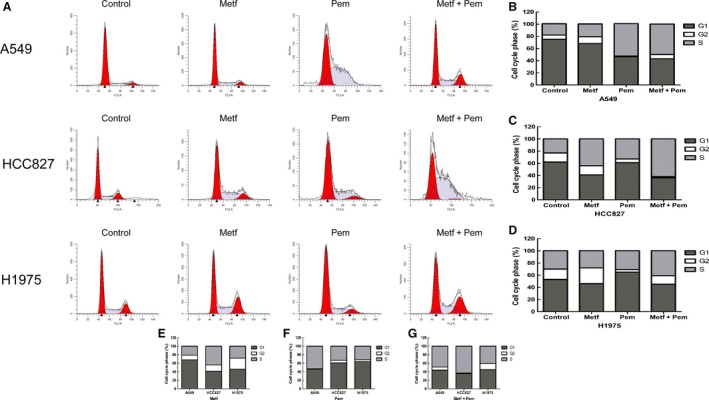
Alteration of the cell cycle distribution of NSCLC cell lines by metformin and (or) pemetrexed. (A) Representative images of the control and different drug groups regarding the cell cycle alteration of the tested cell lines in three independent experiments; (B–D) Alteration of the cell cycle distribution of the A549 (B), HCC827 (C), and H1975 (D) cell lines treated with metformin and (or) pemetrexed compared with that of the control. (E–F) Metformin and (or) pemetrexed showed different effects on the tested NSCLC cell lines.

Cell cycle analysis showed that the three untreated cell lines were all mainly distributed at the G1 phase with 74.75 ± 0.35% for A549, 62.48 ± 1.39% for HCC827, and 53.25 ± 1.88% for H1975, respectively (Table 2). In Figure 3A and B, for the A549 cell line, metformin with IC50 at 48 h did not significantly change the cell cycle distribution compared with the control (*P* > 0.05) and pemetrexed induced marked S phase accumulation of cells with a decreased cell number at the G1 and G2 phases (*P* < 0.0001). The effect of the metformin plus pemetrexed combination on the A549 cell cycle showed no difference compared with that of pemetrexed alone. Metformin caused the S‐phase shift of the HCC827 cell line (*P* < 0.01) together with a decreased cell number at G1 and stable cell number at G2 phase. Pemetrexed also increased the number of HCC827 cells at S phase (*P* < 0.01) but decreased the number at the G2 phase and displayed a stable number at the G1 phase. With the combination of metformin and pemetrexed treatment, the proportion of HCC827 cells in the S phase was markedly increased compared with that of the single drug with a significantly decreased number at both the G1 and S phases (*P* < 0.01 Fig. 3A and C). In Figure 3A and D, with metformin treatment, the proportion of H1975 cells was slightly increased at the G2 phase (*P* < 0.05) with a stable number at the S phase. Additionally, pemetrexed caused G1 phase accumulation together with a stable distribution into the S phase. However, the combined effect of metformin and pemetrexed showed no difference compared with that of pemetrexed alone (Table 2). Above all, the combined treatment of metformin and pemetrexed only strengthened the cell cycle alteration of the HCC827 cell line, but not the A549 and H1975 cell lines (Fig. 3E).

**Table 2:** The each cell cycle distribution of Met or (and) Pem after 48 h (%,_x ± s)

	A549				HCC827				H1975			
	Control	Met	Pem	Met + Pem	Control	Met	Pem	Met + Pem	Control	Met	Pem	Met + Pem
G1	74.75 ± 0.35	68.03 ± 1.70	46.43 ± 3.48	43.54 ± 5.64	62.48 ± 1.39	41.36 ± 0.70	61.41 ± 4.18	36.43 ± 1.28	53.25 ± 1.88*	46.18 ± 6.48	64.83 ± 1.06*	64.80 ± 4.92*^▲^
G2	6.59 ± 1.33	11.36 ± 4.54	0.67 ± 0.65	7.20 ± 5.91	14.66 ± 0.97	15.46 ± 7.7	5.57 ± 1.03	1.09 ± 0.48	17.38 ± 0.69	25.79 ± 4.95*	4.00 ± 3.64	4.41 ± 1.35
S	18.67 ± 1.27	20.69 ± 4.64	53.38 ± 3.44*	51.48 ± 10.99*^▲^	22.86 ± 0.52	43.18 ± 7.07*	33.02 ± 3.32*^#^	62.48 ± 0.97*^▲^	29.37 ± 2.11	28.03 ± 1.54	30.28 ± 5.42	30.79 ± 6.17

A549 cell line: *P < 0.0.0001 compared with the control; ^▲^ *P* > 0.05 compared with the Pem alone;

HCC827 cell line: *P < 0.01 compared with the control; ^#^ *P* > 0.05 compared with the Met alone; ^▲^ *P* < 0.01 compared with the Met or Pem alone

H1975 cell line: *P < 0.05 compared with the control; ^▲^ *P* > 0.05 compared with the Pem alone;

### Inhibition of migration in human NSCLC cell lines

**Figure 4:**
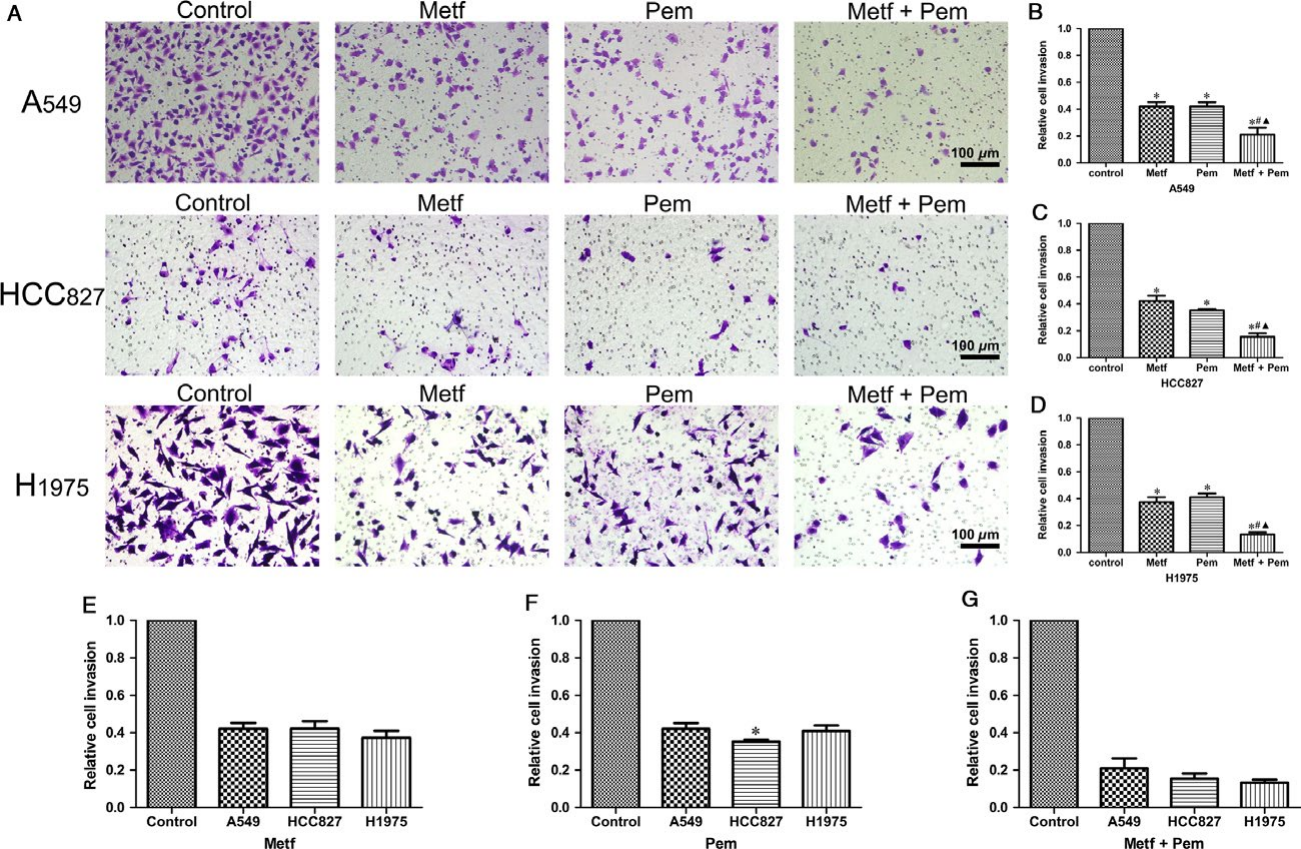
Metformin in combination with pemetrexed could synergistically inhibit the migration ability of NSCLC cell lines. (A) Representative images of the control and different drug groups regarding the migration ability of the tested cell lines in three independent experiments; (B–D) Metformin and (or) pemetrexed could inhibit the migration ability of the A549 (B), HCC827 (C), H1975 (D) cell lines at varying degrees; *P < 0.05 compared with the corresponding control group; ^#^ *P* < 0.05 compared with Metf alone; ^▲^ *P* < 0.05 compared with Pem alone; (E‐F) Regarding the cell lines, no difference was found in the A549 (E) and H1975 (G) cell lines treated with metformin and (or) pemetrexed, whereas the HCC827 cells were more sensitive to pemetrexed alone compared with other two strains, *P < 0.05.

To further determine whether metformin in combination with pemetrexed has a better inhibitory effect on tumor cell migration than metformin or pemetrexed alone, we performed transwell assays. The results showed that metformin or pemetrexed alone with corresponding IC50 at 48 h slightly decreased the migration ability of three NSCLC cell lines (Fig. 4A, E, F). Furthermore, the combination of metformin and pemetrexed enhanced this inhibitory effect compared with that of the mock‐treated groups (*P* < 0.05, Fig. 4A–D, G).

### Regulation of proapoptotic and antiapoptotic markers

**Figure 5:**
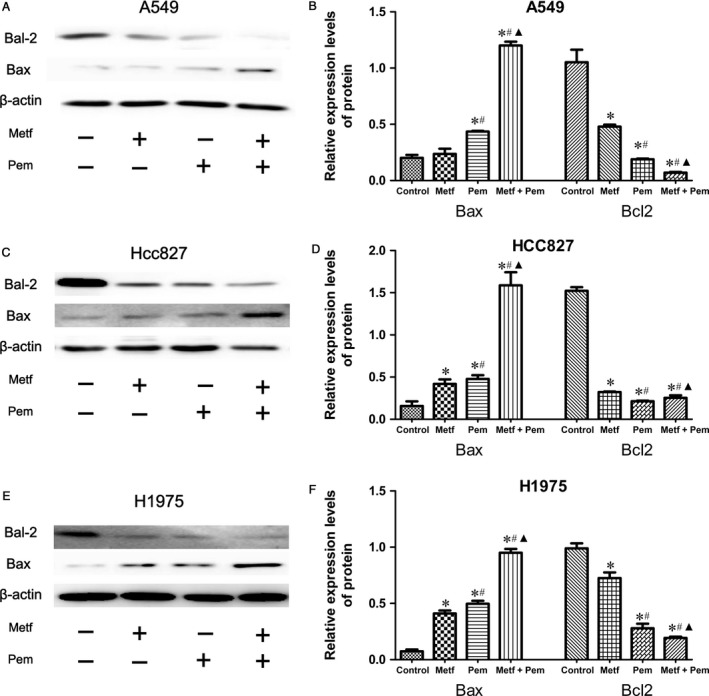
Metformin and pemetrexed alone or the combination could alter the expression level of apoptotic markers in NSCLC cell lines. (A–F) Metformin and pemetrexed alone slightly increased the expression level of bax and decreased the expression level of bcl‐2 in A549 (A), HCC827 (C), and H1975 (E) cells; Metformin in combination with pemetrexed significantly enhanced the effect in A549 (B), HCC827 (D), and H1975 (F) cell lines compared with that of the corresponding single drug groups; *P < 0.05 compared with the corresponding control group; ^#^ *P* < 0.05 compared with Metf alone; ^▲^ *P* < 0.05 compared with Pem alone.

For mammalian cells, Bcl‐2 and Bax, as the main antiapoptotic and proapoptotic target molecules, respectively, in the apoptotic molecular mechanism, were tested in cell‐related experiments. By western blot assay, we found a reduced level of Bcl‐2 and an elevated level of Bax in A549 cells (Fig. 5A and B), HCC827 cells (Fig. 5C and D), and H1975 cells (Fig. 5E and F) after treatment with metformin or pemetrexed alone or both, corresponding to the apoptotic rates of the relevant groups (*P* < 0.05). These results, which were confirmed by western blot analysis, suggested that combined treatment with both metformin and pemetrexed significantly enhanced the apoptotic effect of NSCLC; moreover, metformin‐blocked proliferation and/or survival mechanisms in NSCLC cancer cells might occur via apoptosis.

## Discussion

To verify the antitumor effects of metformin alone or in combination with pemetrexed, we selected the A549, HCC827, and NCI‐H1975 cell lines, which are widely used in the related research of lung adenocarcinoma 20, 21, 22. Additionally, the A549, HCC827, H1975 cell lines represent three main lung adenocarcinoma cell types that harbor wild‐type or various epidermal growth factor receptor (EGFR) mutation genotypes that are associated with sensitivity to tyrosine kinase inhibitors (TKIS) as well as a T790M missense mutation with acquired drug‐resistance to EGFR‐TKIs to reflect the antitumor effect of metformin or be combined with chemotherapy to be used as a comprehensive treatment for lung adenocarcinoma cell lines.

In the research of Zhao ZQ, et al., the concentration of metformin in the A549 and HCC827 cell lines was 1–10 mmol/L, which exerted a significant inhibition on cell proliferation and invasion ability 21. Moreover, Bianca Sperl et al. performed an experiment concerning metformin and salinomycin in the treatment of NSCLC and obtained mean IC50 values of metformin of approximately 2.5 mmol/L and 5 mmol/L for HCC4006 and NCI‐H1975 cells, respectively 23. Moreover, Yukihisa Hatakeyama, et al. used a series of concentration ranging from 0 to 25.6 *μ*mol/L and detected the IC50 (1.6 *μ*mol/L) of pemetrexed for A549 cells 24. Another study by Chi LY et al. also described the IC50 of pemetrexed for A549 cells (1304.7 ± 94.7 nmol/L) 25. The concentration range of metformin and pemetrexed used in our study, as previously indicated, is in accordance with the results of the preliminary experiment above.

In this study, we found that metformin alone can inhibit the proliferation, invasion ability and induction of apoptosis in the tested cells, with a concentration ranging from 5 mmol/L to 20 mmol/L. In addition, metformin in combination with pemetrexed could only alter the cell cycle distribution of the HCC827 cell line, but not that of the A549 and H1975 cell lines. More importantly, metformin in combination with pemetrexed had synergistic effects in the treatment of NSCLC in vitro. Through the detection of cell apoptosis markers Bcl‐2 and Bax in the treated or control groups and a data comparison between the different groups, the synergistic effects between metformin and pemetrexed on NSCLC or sensitization effect of metformin to chemotherapy were verified again. Our data demonstrated the relationship between metformin and pemetrexed in treating NSCLC in vitro, which is consistent with the previous findings regarding metformin and other chemicals, such as cisplatin or etoposide 18, 19.

Pem combined with cisplatin or carboplatin was recommended as a first‐line treatment for the patients with NSCLC and Pem alone can be as maintenance therapy 26, 27. Pemetrexed is a folate antimetabolite drug that works by inhibiting three enzymes used in purine and pyrimidine synthesis–thymidylate synthase (TS), which is necessary for DNA replication and repair; dihydrofolate reductase (DHFR); and glycinamide ribonucleotide formyltransferase (GARFT) 28, 29. The mechanisms of the antitumor effects of metformin have been investigated by many researchers, and we present the following summary: (1) Metformin can activate AMP‐activated protein kinase (AMPK) mediated by liver kinase B1 (LKB1) 30, leading to the suppression of mammalian target of rapamycin (mTOR) signaling, which is an essential inhibitor of nutrient signals and growth factors, and inhibition of protein synthesis and cancer cell proliferation 31; (2) Metformin could increase the activity of p53 and consequently arrest the cell cycle in G1 phase 32; (3) Metformin might promote cancer cell apoptosis via the mitogen‐activated protein kinase (MAPK) signaling pathway and upregulate growth arrest and DNA damage153(GADD153),which is an apoptosis‐related gene 16; and (4) Metformin could enhance the curative effect of chemotherapy or increase the sensitivity of cancer cells to chemotherapy. Moreover, there may be other mechanisms, such as inhibiting epithelial‐mesenchymal transformation (EMT) or specific effects on residual cells or tumor stem cells to inhibit cancer cell growth 22, 33, 34.

According to our experimental data, metformin in combination with pemetrexed showed greater inhibition of proliferation as well as increased invasion ability on lung adenocarcinoma cancer cells in vitro and a higher apoptosis rate, activities that were validated by apoptosis‐related biomarkers. We speculate that the possible mechanism for the synergistic effect of metformin combined with chemotherapy is related to apoptotic cell death activation. The significant downregulated expression of Bcl‐2, which is an antiapoptotic marker, and upregulated expression of the proapoptotic marker Bax in cultured NSCLC cell lines as detected by western blotting suggested that the common apoptotic pathways were activated and enhanced apoptotic cancer cell death. Our points of view are in accordance with those of Y Storozhuk et al. 14. Moreover, metformin and (or) pemetrexed did not cause cell cycle alterations in all of the tested cell lines, indicating that the synergistic effect on NSCLC cells might not be from blocking the cell cycle of the tested cells, a finding that requires deep investigation to confirm.

The concentration of metformin in our study and others (2–20 mmol/L) 16, 22 is approximately 100‐fold higher than that of the mean peak plasma in clinical available diabetes patients (15 × 10^‐3^ mmol/L with the maximal dose of 750 mg of metformin). The results from basic studies cannot be directly applied to clinical cancer treatment, but there is still certain clinical significance that shows that: (1) Long‐term use of metformin may enhance and amplify its antitumor effect similar to that in an in vitro experiment; (2) Accumulation metformin in tissue is more than that in the blood; (3) Tolerance and cytotoxicity of metformin in combination with chemotherapy for cancer treatment may be improved; and (4) Metformin as a small molecule is quickly pumped out of cells and is difficult to accumulate in tissue at a low concentration. To further elucidate the antitumor effects of metformin in combination with chemicals, more in‐depth investigation is needed in joint laboratory studies regarding its clinical use.

In conclusion, our data demonstrate that metformin in combination with pemetrexed shows greater inhibition of proliferation, invasion, migration and induction of apoptosis in NSCLC cell lines than metformin or pemetrexed used as monotherapy. Additionally, this action may be due to the enhanced proapoptotic effects of the combined therapy or blockage of the cell cycle or the conventional LKB1‐AMPK‐mTOR signaling pathway. The synergistic effect of metformin and pemetrexed or chemosensitization of metformin provides a promising treatment option and preclinical basis for patient treatment for NSCLC.

## Conflict of Interest

None declared.
